# Intracellular Ca^2+^-Mediated Mechanisms for the Pacemaker Depolarization of the Mouse and Guinea Pig Sinus Node Tissue

**DOI:** 10.3390/biom12030377

**Published:** 2022-02-28

**Authors:** Iyuki Namekata, Kento Jitsukata, Ayumi Fukuda, Ryosuke Odaka, Shogo Hamaguchi, Hikaru Tanaka

**Affiliations:** Department of Pharmacology, Faculty of Pharmaceutical Sciences, Toho University, 2-2-1 Miyama Funabashi, Chiba 274-8510, Japan; kento.jitsukata@gmail.com (K.J.); 1015188f@st.toho-u.jp (A.F.); 3021002o@st.toho-u.jp (R.O.); shogo.hamaguchi@phar.toho-u.ac.jp (S.H.); htanaka@phar.toho-u.ac.jp (H.T.)

**Keywords:** cardiac pacemaking, Na^+^-Ca^2+^ exchanger, pacemaker depolarization, sinus node

## Abstract

Intracellular Ca^2+^-mediated mechanisms for pacemaker depolarization were studied in sinus node tissue preparations from mice and guinea pigs. Microelectrode recordings revealed that the sinus node of the mouse, which had a higher beating rate, had a steeper slope of the pacemaker depolarization than that of the guinea pig. BAPTA and ryanodine, agents that interfere with intracellular Ca^2+^, significantly decreased the slope of the pacemaker depolarization in both species. In contrast, SEA0400, a specific inhibitor of the Na^+^-Ca^2+^ exchanger (NCX), as well as change to low Na^+^ extracellular solution, significantly decreased the slope in the mouse, but not in the guinea pig. Niflumic acid, a blocker of the Ca^2+^ activated Cl^−^ channel, decreased the slope in both species. Confocal microscopy revealed the presence of spontaneous Ca^2+^ oscillations during the interval between Ca^2+^ transients; such phenomenon was more pronounced in the mouse than in the guinea pig. Thus, although intracellular Ca^2+^-mediated mechanisms were involved in the pacemaker depolarization of the sinus node in both species, the NCX current was involved in the mouse but not in the guinea pig.

## 1. Introduction

The contraction of the myocardium is driven by the action potential originating in the sinus node, the cardiac pacemaker. The action potential of the sinus node has a characteristic pacemaker depolarization phase (phase 4 depolarization) in the membrane voltage range of −60–−40 mV which leads the membrane potential to the threshold level for the rapid action potential upstroke (phase 0 depolarization). The pacemaker depolarization is formed by multiple inward membrane currents including the hyperpolarization-activated current (I_f_), L-type and T-type Ca^2+^ currents, and the sustained inward current (I_st_) [1,2,3,4,5]. The combination of membrane currents involved in the pacemaker depolarization appears to be different among animal species. For example, the I_f_ current manifests in sinus node cells of most species including mice, guinea pigs, and rabbits, while it was masked by the inwardly rectifying K^+^ current (I_K1_) in rats and monkeys [6]. The current density and function of the T-type Ca^2+^ current was reported to be larger in the sinus nodes from smaller animals [3,7]. 

It has been postulated that the pacemaker depolarization is also influenced by intracellular Ca^2+^. The Ca^2+^ released from the sarcoplasmic reticulum (SR) causes the Na^+^-Ca^2+^ exchanger (NCX) to generate a depolarizing current and accelerate the pacemaker depolarization. This mechanism was referred to as the Ca^2+^ clock and was considered to function in coordination with the ion channels of the cell membrane (membrane clock) to form the pacemaker depolarization [8,9]. However, the related experimental results appear to vary among researchers, and definitive conclusions have not been reached for the involvement of intracellular Ca^2+^-mediated mechanisms or the precise pacemaking mechanisms in each animal species. For example, in the guinea pig sinus node, some researchers emphasize the involvement of the Ca^2+^-clock while others reported negative results [4,9,10].

One of the possible reasons for this discrepancy is the use of isolated sinus node cells. The sinus node consists of cardiomyocytes with different electrophysiological properties [5,11], and these cells work as a functional syncytium to form the pacemaker depolarization. Thus, in a strict sense, none of the cells isolated from the sinus node region exactly represent the sinus node pacemaker. Thus, it is of value to obtain information on the function of the sinus node as a whole. In the present study, we intended to clarify the involvement of intracellular Ca^2+^-mediated mechanisms in the pacemaker depolarization of the mouse and guinea pig sinus node using tissue preparations. We performed standard microelectrode measurements of action potentials with sinus node tissue preparations and applied selective pharmacological agents.

## 2. Materials and Methods

Standard glass microelectrode experiments were performed with isolated sinus node tissue from the mouse and the guinea pig as described previously [2,3]. Microelectrode penetrations into the sinus node region were made from the epicardial surface. The extracellular solution was of the following composition (mM concentration): NaCl 118.4, KCl 4.7, CaCl_2_ 2.5, MgSO_4_ 1.2, KH_2_PO_4_ 1.2, NaHCO_3_ 24.9, and glucose 11.1 (pH 7.4), and the solution was gassed with 95% O_2_-5% CO_2_ and maintained at 36 ± 0.5 °C. The low Na^+^ extracellular solution was prepared with the equimolar substitution of NaCl with LiCl so that the final Na^+^ concentration was 70 mM. Change to low Na^+^ solution and return to normal solution were performed with a flow-switching device which enabled change of the extracellular solution within approximately 1 s. To chelate intracellular Ca^2+^ with O,O’-Bis (2-aminophenyl) ethyleneglycol-N,N,N′,N′-tetraacetic acid (BAPTA), its cell-permeable tetraacetoxymethyl ester (BAPTA-AM) was applied to the preparations at a final concentration of 300 μM.

The action potential parameters measured were firing rate, cycle length, maximum diastolic potential, threshold potential, the slope of the pacemaker depolarization (slope), maximum rate of rise of the action potential upstroke (maximum rate of rise; (dV/dt)_max_), peak potential, and duration at 50% repolarization (APD_50_). To obtain the slope value, the middle portion of the pacemaker depolarization phase was fitted by a straight line; the curved regions close to the maximum diastolic potential and threshold potential were not included in the fitting. The spontaneous firing of the sinus node tissue preparations was well maintained; the change in firing rate at 10 and 30 min after the addition of vehicle was less than 1% and 2% of the basal value, respectively.

For the analysis of intracellular Ca^2+^ movements, the sinus node tissue preparations were incubated with a Ca^2+^ indicator (Cal-590 AM) at 10 μM for 1 h at 37 °C in the following composition: NaCl 143, KCl 4.7, MgCl_2_ 1.0, NaH_2_PO_4_ 0.33, glucose 5.5, and HEPES 5 (mM). The recording chamber, the bottom of which was a coverslip, was placed on the stage of an inverted microscope. Preparations were placed at the bottom of the recording chamber epicardial surface down. The normal extracellular solution (NaCl 143, KCl 4.7, MgCl_2_ 1.0, CaCl_2_ 1.8, NaH_2_PO_4_ 0.33, glucose 5.5, and HEPES 5 (mM) maintained at 32 °C) was continuously perfused. Confocal microscopic analyses of the Ca^2+^ oscillations in the myocardial layer were performed with a rapid scanning confocal microscope A1R (Nikon, Tokyo, Japan). The emission 570–620 nm on excitation at 561 nm was detected for Cal-590 fluorescence, and the scanning was performed at a speed of frame/16.9–33.8 ms. The fluorescent intensity of Cal-590 at each time point was normalized against the basal intensity.

BAPTA-AM (Tokyo Chemical Industry, Tokyo, Japan), ryanodine (Wako Pure Chemical Industries, Osaka, Japan), SEA0400 (synthesized in our faculty), niflumic acid (Sigma-Aldrich, St. Louis, MO, USA), and Cal-590 AM (Cosmo Bio, Tokyo, Japan) were dissolved in dimethyl sulfoxide (DMSO). All data were expressed as the mean ± standard error of the mean (S.E.M). Data were analyzed by the paired *t*-test. A *p* value less than 0.05 was considered statistically significant.

## 3. Results

Microelectrode recordings showed that the sinus node of the mouse, which had a higher firing rate, had a steeper slope of the pacemaker depolarization and a shorter action potential duration than that of the guinea pig. There was no difference in the maximum diastolic potential and threshold potential between the mouse and guinea pig; the maximum diastolic potential was about −60 mV, and the threshold potential was about −40 mV in both species.

**Figure 1:**
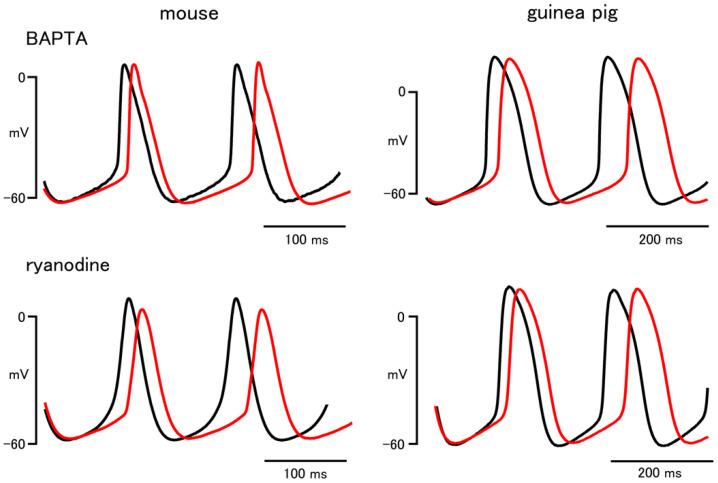
Effects of BAPTA and ryanodine on the sinus node action potential of the mouse and guinea pig. Typical traces before (black lines) and after (red lines) application of 300 μM BAPTA (upper) and 0.1 μM ryanodine (lower).

In the mouse sinus node, chelation of intracellular Ca^2+^ with BAPTA (300 μM) induced significant decreases in the firing rate and the slope of the pacemaker depolarization (Figure 1 and Table 1). BAPTA had no significant effect on other parameters in the mouse. In the guinea pig sinus node, BAPTA induced significant decreases in the firing rate and the slope of the pacemaker depolarization (Figure 1 and Table 1). BAPTA shifted the maximum diastolic potential and the threshold potential towards depolarized potentials in the guinea pig.

In the mouse and guinea pig sinus node, ryanodine (0.1 μM) significantly decreased the firing rate and the slope of the pacemaker depolarization (Figure 1 and Table 1). Ryanodine had no significant effect on other parameters in both species.

**Figure 2:**
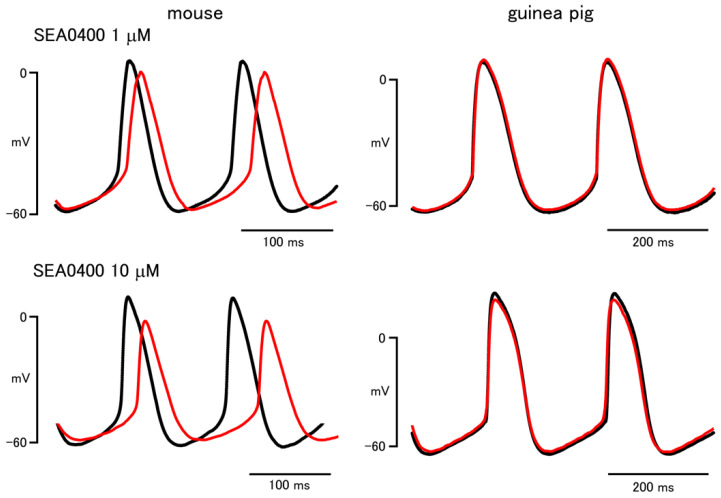
Effect of SEA0400 on the sinus node action potential of the mouse and guinea pig. Typical traces before (black lines) and after (red lines) application of 1 μM (upper) and 10 μM (lower).

In the mouse sinus node, SEA0400 (1 and 10 μM) decreased the firing rate and the slope of the pacemaker depolarization (Figure 2 and Table 2). SEA0400 shifted the maximum diastolic potential and the threshold potential towards depolarized potentials and decreased the maximum rate of rise and the peak potential in the mouse. Further, when the effect of SEA0400 on the beating rate of the mouse right atria was examined after the beating rate was decreased with carbachol, SEA0400 significantly decreased the beating rate; the beating rate in the presence of 0.3 μM carbachol before and after the application of SEA0400 (1 μM) was 234.1 ± 10.7 and 163.8 ± 29.1 (*n* = 6), respectively.

In the guinea pig sinus node, SEA0400 had no effect on the action potential waveform (Figure 2 and Table 2). Further, when the effect of SEA0400 on the beating rate of the guinea pig right atria was examined after the beating rate was increased with noradrenaline, SEA0400 had no effect on the beating rate; the beating rate in the presence of 3 μM noradrenaline before and after the application of SEA0400 (1 μM) was 328.4 ± 8.9 and 324.6 ± 8.9 (*n* = 5), respectively.

**Figure 3:**
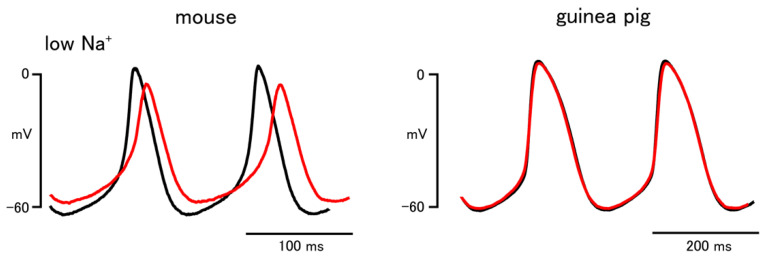
Effect of low Na^+^ solution on the sinus node action potential of the mouse and guinea pig. Typical traces before (black lines) and after (red lines) rapid change of the extracellular solution to low Na^+^ solution.

To support the results obtained with SEA0400, we intended to reduce the inward NCX current with low Na^+^ extracellular solution. In the mouse sinus node, changing the extracellular solution to low Na^+^ solution significantly decreased the firing rate and the slope of the pacemaker depolarization (Figure 3 and Table 3). The low Na^+^ solution shifted the maximum diastolic potential and the threshold potential towards depolarized potentials and decreased the maximum rate of rise and the peak potential. In the guinea pig, changing the extracellular solution to low Na^+^ solution had no significant effect on the firing rate and the slope of the pacemaker depolarization. The low Na^+^ solution did not affect any of the action potential parameters (Figure 3 and Table 3).

**Figure 4:**
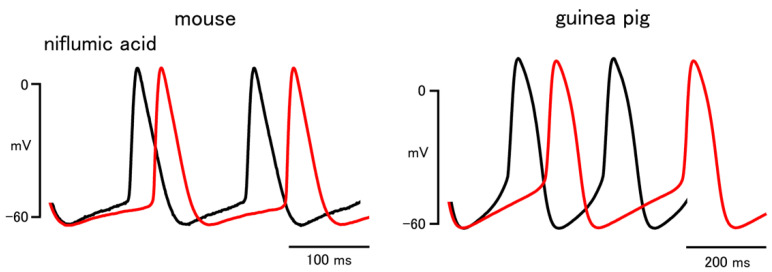
Effect of niflumic acid on the sinus node action potential of the mouse and guinea pig. Typical traces before (black lines) and after (red lines) application of 30 μM niflumic acid.

In the mouse sinus node, niflumic acid (30 μM), a blocker of the Ca^2+^-activated Cl^−^ channel, significantly decreased the firing rate and the slope of the pacemaker depolarization (Figure 4 and Table 4). Niflumic acid had no significant effect on other parameters in the mouse. In the guinea pig sinus node, niflumic acid significantly decreased the firing rate and the slope of the pacemaker depolarization (Figure 4 and Table 4). The action potential duration was shortened by niflumic acid in the guinea pig.

**Figure 5:**
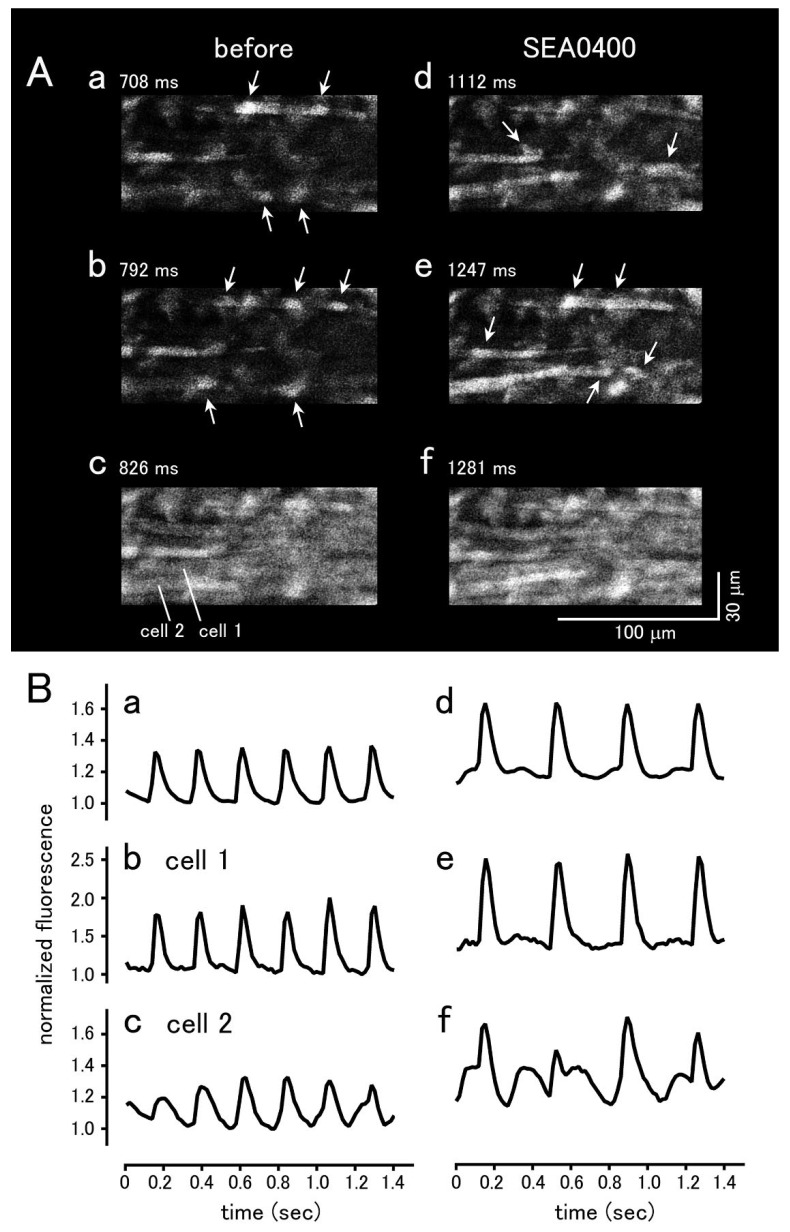
Effect of SEA0400 on spontaneously occurring Ca^2+^ transients in the mouse sinus node tissue. (**A**) Typical x-y images of the myocardium loaded with Cal-590 before (**a**–**c**) and 10 min after the addition of 1μM SEA0400 (**d**–**f**). Panels c and f are the images at the peak of the Ca^2+^ transient. Note that Ca^2+^ oscillations were observed during the interval between the Ca^2+^ transients (arrows). (**B**) Time course of the changes in fluorescence before addition (**a**–**c**) and 10 min after the addition of 1 μM SEA0400 (**d**–**f**). Time course of the fluorescence intensity quantified in the whole field of view (**a**,**d**), cell 1 (**b**,**e**), and cell 2 (**c**,**f**) as shown in panel (**A**)/(**c**).

**Figure 6:**
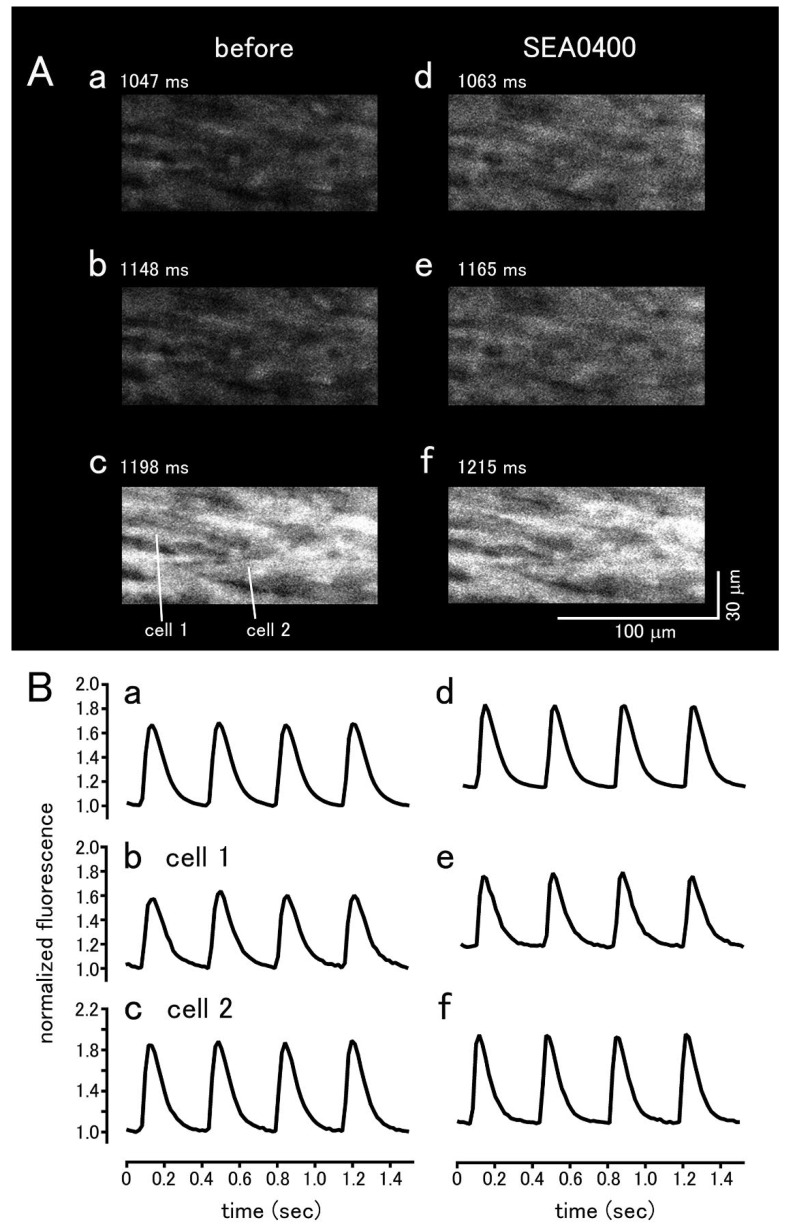
Effect of SEA0400 on spontaneously occurring Ca^2+^ transients in the guinea pig sinus node tissue. (**A**) Typical x-y images of the myocardium loaded with Cal-590 before (**a**–**c**) and 10 min after the addition of 1μM SEA0400 (**d**–**f**). Panels c and f are the images at the peak of the Ca^2+^ transient. Note that no Ca^2+^ oscillation was observed during the interval between the Ca^2+^ transients (**a**,**b**,**d**,**e**). (**B**) Time course of the changes in fluorescence before addition (**a**–**c**) and 10 min after the addition of 1 μM SEA0400 (**d**–**f**). Time course of the fluorescence intensity quantified in the whole field of view (**a**,**d**), cell 1 (**b**,**e**), and cell 2 (**c**,**f**) as shown in panel (**A**)/(**c**).

To analyze intracellular Ca^2+^ movements, the sinus node tissue preparations loaded with the Ca^2+^ sensitive fluoroprobe, Cal-590, were observed with a rapid scanning confocal microscope. In both species, Ca^2+^ transients, synchronized elevations of Ca^2+^ fluorescence throughout the myocardial tissue, were observed at a constant frequency. In mouse sinus node, spontaneous Ca^2+^ oscillations, non-synchronized local rises in Ca^2+^ fluorescence, were observed in roughly one third of the cells (Figure 5). Such Ca^2+^ oscillations were less frequently observed in the guinea pig (Figure 6).

In the mouse sinus node, SEA0400 (1 μM) markedly reduced the firing frequency of spontaneous Ca^2+^ transients (Figure 5); the frequency before and 10 min after the addition of 1 μM SEA0400 was 185.8 ± 24.1 and 110.3 ± 23.2 bpm (*n* = 5; *p* < 0.05), respectively. SEA0400 caused an increase in basal Ca^2+^ concentration; the basal Ca^2+^ fluorescence at 10 min after the addition of 1 μM SEA0400 was 110.3 ± 8.7% (*n* = 5) of the value before application. SEA0400 had no apparent effect on the local Ca^2+^ oscillations.

In the guinea pig sinus node, SEA0400 had no effect on the firing frequency of spontaneous Ca^2+^ transients (Figure 6); the frequency before and 10 min after the addition of 1 μM SEA0400 was 157.2 ± 5.9 and 158.2 ± 8.5 bpm (*n* = 5), respectively. SEA0400 caused an increase in basal Ca^2+^ concentration; the basal Ca^2+^ fluorescence at 10 min after the addition of 1 μM SEA0400 was 112.0 ± 4.5% (*n* = 5) of the value before application.

## 4. Discussion

In the present study, we intended to clarify the involvement of intracellular Ca^2+^-mediated mechanisms in the pacemaker depolarization of the mouse and guinea pig sinus node. We applied standard microelectrode techniques on tissue preparations and used selective pharmacological agents. Compared to isolated cardiomyocytes, myocardial tissue preparations may have disadvantages such as less efficient delivery of oxygen and pharmacological agents to the cells of interest. On the other hand, they have advantages such as absence of cell isolation damage and cell selection bias. Above all, tissue preparations enable the observation of the sinus node as a whole working as a functional syncytium.

First of all, we examined whether or not intracellular Ca^2+^-mediated mechanisms are involved in the pacemaking of the sinus node. Treatment of the sinus node tissue preparations with either BAPTA or ryanodine, agents that interfere with intracellular Ca^2+^, decreased the firing rate and the slope of the pacemaker depolarization both in the mouse and guinea pig. These results indicated that Ca^2+^ released from the SR through the ryanodine receptor contributes to the formation of the pacemaker depolarization of the sinus node in both species. As both BAPTA and ryanodine may have a broad effect on intracellular Ca^2+^-mediated events, their effects may also reflect changes other than the instantaneous reduction of intracellular Ca^2+^ [4]. Thus, we intended to clarify the transporters that convert intracellular Ca^2+^ into the pacemaker depolarization.

The Na^+^/Ca^2+^ exchanger (NCX) is the major pathway for transsarcolemal Ca^2+^ extrusion from the cytoplasm; it pumps out one Ca^2+^ ion in exchange for three Na^+^ ions (forward mode NCX). It has been postulated that the net inward current that occurs in this process contributes to the pacemaker depolarization of the sinus node. To examine this hypothesis in sinus node tissue preparations, we used SEA0400, a potent and selective inhibitor of NCX. In voltage-clamped guinea pig ventricular myocytes, 1 μM SEA0400 inhibited the NCX current by more than 80%, with no effect on the sodium current, L-type calcium current, delayed rectifier potassium current, and the inwardly rectifying potassium current [12]. The effectiveness of SEA0400 on tissue preparations was also confirmed in the mouse and guinea pig myocardium [13,14,15].

In the mouse sinus node, the slope of the pacemaker depolarization, as well as the firing rate of action potentials and Ca^2+^ transients, was decreased by SEA0400. Similar results were obtained with low Na^+^ extracellular solution. Thus, the NCX current activated by the Ca^2+^ released from the SR contributes to the pacemaker depolarization in mouse sinus node. These results were consistent with the observation that the firing frequency of the sinus node was lower in atrial-specific NCX knock out mice than in wild type mice [16]. In contrast, in the guinea pig sinus node, treatment with SEA0400, as well as low Na^+^ extracellular solution, affected neither the firing rate nor the slope of the pacemaker depolarization. SEA0400 caused a small increase in the basal Ca^2+^ concentration. These results indicated that NCX contributes to transarcolemmal Ca^2+^ efflux but does not play an essential role in the pacemaker depolarization of the guinea pig sinus node. This lack of SEA0400 effect in the guinea pig sinus node was not due to its lower firing rate because SEA0400 was ineffective in the guinea pig right atria even after its beating rate was increased by noradrenaline to a level similar to that of the mouse, and effective in the mouse right atria even after its beating rate was decreased by carbachol to a level similar to that of the guinea pig.

The effects of SEA0400 and low Na^+^ on the maximum rate of rise and the peak potential could be explained by their effect on the pacemaker depolarization. SEA0400 and low Na^+^ markedly reduced the slope of pacemaker depolarization in the mouse sinus node. This means that the membrane potential is kept in a partially depolarized potential range close to the threshold potential for a longer time, which may cause increased inactivation of the L-type Ca^2+^ channel leading to a reduced maximum rate of rise and peak potential. In contrast, in the guinea pig sinus node, in which the slope of the pacemaker depolarization was unchanged, neither the maximum rate of rise nor the peak potential was affected.

The pacemaker depolarization is considered to be formed by several inward currents such as the hyperpolarization-activated current (I_f_), L-type and T-type Ca^2+^ currents, and the sustained inward current (I_st_) [1,2,3,4,5]. In addition, the deactivation of I_K_ is dominant in the early phase of pacemaker depolarization in the guinea pig sinus node [4,6]. The NCX current in the sinus node was detected both in the mouse and guinea pig; the current density was not different between the two species under voltage-clamp conditions [17,18]. Since the NCX currents observed were relatively small (<5 pA/pF), they may possibly be masked by other membrane currents. As the NCX current density in the intact sinus node is affected by the intracellular supply of Ca^2+^ to the NCX, it is possible that some difference in intracellular Ca^2+^ handling properties causes a difference in NCX current density in the spontaneously firing sinus node. In fact, spontaneous Ca^2+^ oscillations were observed during the interval between Ca^2+^ transients in sinus node tissue preparations, which was more pronounced in the mouse than in the guinea pig. This appears to be consistent with the observation in isolated sinus node cardiomyocytes that the number of Ca^2+^ sparks occurring during the pacemaker depolarization was larger in the mouse than in the guinea pig [9,19].

Concerning the sinus node of other animal species, it was reported that SEA0400 had no effect on the heart rate in the isolated rabbit heart [20] and in anesthetized dogs [21]. To the best of our knowledge, there is no information on the effect of SEA0400 on the human sinus node. Concerning ectopic pacemakers, we observed in the guinea pig pulmonary vein that both SEA0400 and ryanodine inhibited the spontaneous electrical activity [22]. Ca^2+^ sparks occurred during the interval between Ca^2+^ transients, and the frequency of Ca^2+^ spark firing increased abruptly just before the onset of Ca^2+^ transients [23]. These results indicated that the NCX current activated by Ca^2+^ released from the SR was involved in the pacemaker depolarization of the pulmonary vein myocardium. Thus, the contribution of NCX to myocardial pacemaking varies among animal species and type of myocardium.

To obtain further information on the Ca^2+^-mediated pacemaking mechanisms of the sinus node, we examined the effect of niflumic acid, a blocker of the Ca^2+^-activated Cl^−^ channel, which was identified in sinus node cells [24,25]. As the estimated reversal potential for Cl^−^ ions in cardiomyocytes was about −30 mV [26], activation of the Ca^2+^-activated Cl^−^ channel could generate a depolarizing current during the pacemaker depolarization. Niflumic acid reduced the firing rate and the slope of pacemaker depolarization in the sinus node of both mouse and guinea pig. These results indicated that the inward current through the Cl^−^ channel activated by Ca^2+^ released from the SR contributes to the pacemaker depolarization of the sinus node both in the mouse and guinea pig.

The mouse has a high heart rate (400 bpm>), and its sinus node has a steeper slope of pacemaker depolarization among experimental animal species. The present study showed that the Ca^2+^-activated Cl^−^ current (I_ClCa_) was involved in the pacemaker depolarization of both mouse and guinea pig, but the NCX current contributed to cardiac pacemaking only in the mouse. Using similar experimental techniques, we previously showed that the contribution of the T-type Ca^2+^ channel to cardiac pacemaking differs among animal species; the effects of *R*-(−)-efonidipine, which is a selective T-type Ca^2+^ channel inhibitor, was prominent in the mouse, small but significant in the guinea pig, and was not observed in the rabbit [2]. Voltage gated Na^+^ channels, which are considered not to play a role in pacemaker activity in the majority of mammalian species including the guinea pig [10], was reported to play a certain role in the pacemaking of the mouse sinus node [27]. The present and earlier studies revealed that the guinea pig sinus node depends on I_CaL_, I_st_, I_f_, and I_ClCa_, but not I_NCX_ for its pacemaking [4,5,11]. In the mouse sinus node, additional currents including the I_CaT_, I_NCX_, and I_Na_ also contribute to pacemaking [2,7,16,27]. Thus, the mouse sinus node appears to have a greater variety of ionic mechanisms to maintain its high firing rate.

## 5. Conclusions

Intracellular Ca^2+^-mediated mechanisms were involved in the pacemaker depolarization of the sinus node both in the mouse and guinea pig. The NCX current was involved in the mouse but not in the guinea pig. This difference could partly explain the higher firing rate of the mouse sinus node.

## Figures and Tables

**Table 1:** Action potential parameters before and after application of BAPTA and ryanodine.

	BAPTA		Ryanodine	
	Mouse	Guinea Pig	Mouse	Guinea Pig
Firing rate (bpm)	423.7 ± 10.1	241.4 ± 16.1	453.6 ± 10.0	248.7 ± 11.7
	396.0 ± 6.4 *	213.1 ± 12.2 *	414.3 ± 13.9 *	237.4 ± 12.6 *
Cycle length (ms)	141.9 ± 3.4	254.2 ± 17.0	132.5 ± 2.9	241.3 ± 13.4
	151.7 ± 2.4 *	286.7 ± 17.2 *	145.5 ± 4.7 *	256.7 ± 13.6 *
Maximum diastolic potential (mV)	−60.6 ± 1.4	−62.9 ± 1.2	−58.8 ± 1.2	−63.2 ± 1.3
	−60.0 ± 1.7	−58.9 ± 2.1 *	−55.9 ± 1.7	−60.9 ± 1.7
Slope of pacemaker depolarization (mV/s)	192.0 ± 26.2	147.4 ± 14.7	223.5 ± 25.8	138.2 ± 10.4
	130.0 ± 17.7 *	104.8 ± 9.8 *	110.0 ± 24.5 *	119.2 ± 9.1 *
Threshold potential (mV)	−50.4 ± 1.6	−49.0 ± 1.6	−46.9 ± 1.9	−52.6 ± 1.9
	−51.6 ± 1.5	−46.4 ± 2.1 *	−45.8 ± 3.0	−49.1 ± 2.3
Maximum rate of rise (V/s)	10.3 ± 2.6	9.0 ± 2.2	8.7 ± 2.7	18.7 ± 9.4
	9.0 ± 2.2	8.1 ± 1.9	7.8 ± 2.2	15.6 ± 7.8
Peak potential (mV)	0.9 ± 2.5	18.9 ± 1.1	4.6 ± 1.2	18.3 ± 1.7
	−0.9 ± 3.2	17.7 ± 1.5	4.2 ± 1.0	17.8 ± 1.6
Duration at 50% repolarization (ms)	30.3 ± 1.7	81.8 ± 2.0	28.3 ± 0.9	75.4 ± 5.9
	30.2 ± 1.4	85.6 ± 6.3	28.9 ± 1.4	77.4 ± 6.4

Upper and lower values in each row indicate the parameters obtained before and after (10 min for BAPTA and 5 min for ryanodine) the application of agents, respectively. Values indicate the mean ± S.E.M. from 5–6 preparations. Asterisks indicate significant difference from corresponding values before the application of agents as evaluated by the paired *t*-test (*p* < 0.05).

**Table 2:** Action potential parameters before and after application of SEA0400.

	SEA0400 (1 μM)		SEA0400 (10 μM)	
	Mouse	Guinea Pig	Mouse	Guinea Pig
Firing rate (bpm)	449.7 ± 24.9	239.0 ± 9.2	428.2 ± 27.2	239.4 ± 8.9
	396.8 ± 29.0 *	240.1 ± 9.2	363.8 ± 17.7 *	237.4 ± 11.6
Cycle length (ms)	135.2 ± 8.1	253.0 ± 10.1	142.1 ± 8.0	252.0 ± 9.0
	155.0 ± 12.2 *	251.8 ± 9.7	166.4 ± 7.6 *	255.1 ± 12.3
Maximum diastolic potential (mV)	−55.8 ± 1.8	−62.6 ± 2.5	−59.2 ± 1.0	−61.7 ± 2.1
	−51.7 ± 1.9 *	−62.1 ± 2.4	−55.9 ± 1.3 *	−59.1 ± 3.0
Slope of pacemaker depolarization (mV/s)	217.0 ± 22.2	148.3 ± 16.2	215.4 ± 26.6	172.5 ± 6.0
	173.2 ± 22.9 *	150.8 ± 13.8	161.0 ± 20.9 *	177.0 ± 16.3
Threshold potential (mV)	−46.0 ± 1.2	−49.8 ± 2.7	−48.6 ± 1.2	−48.3 ± 2.5
	−41.9 ± 1.4 *	−48.7 ± 2.5	−45.5 ± 1.3 *	−43.9 ± 3.1
Maximum rate of rise (V/s)	6.7 ± 0.6	7.0 ± 1.5	7.9 ± 1.6	5.8 ± 1.5
	5.6 ± 0.7 *	7.7 ± 2.2	4.5 ± 0.8 *	4.8 ± 1.1
Peak potential (mV)	4.8 ± 0.7	12.0 ± 2.1	4.7 ± 1.6	11.9 ± 1.6
	−0.5 ± 1.3 *	12.8 ± 1.8	−11.2 ± 3.0 *	12.0 ± 1.6
Duration at 50% repolarization (ms)	33.5 ± 2.1	80.4 ± 5.4	31.3 ± 2.2	85.4 ± 6.2
	35.5 ± 1.5	79.8 ± 4.4	36.2 ± 2.2	83.6 ± 5.3

Upper and lower values in each row indicate the parameters obtained before and after (10 min) the application of agents, respectively. Values indicate the mean ± S.E.M. from 5–6 preparations. Asterisks indicate significant difference from corresponding values before the application of agents as evaluated by the paired *t*-test (*p* < 0.05).

**Table 3:** Action potential parameters before and after the rapid change of the extracellular solution to low Na^+^ solution.

	Low Na^+^	
	Mouse	Guinea Pig
Firing rate (bpm)	498.4 ± 17.1	232.7 ± 9.5
	455.5 ± 20.5 *	232.7 ± 9.9
Cycle length (ms)	121.1 ± 4.0	260.2 ± 12.0
	133.1 ± 5.9 *	260.5 ± 12.6
Maximum diastolic potential (mV)	−60.4 ± 1.1	−63.2 ± 0.9
	−56.9 ± 0.9 *	−64.0 ± 1.6
Slope of pacemaker depolarization (mV/s)	251.4 ± 25.3	166.3 ± 17.4
	206.7 ± 30.7 *	187.3 ± 20.4
Threshold potential (mV)	−49.8 ± 1.1	−48.2 ± 1.1
	−47.6 ± 1.0 *	−48.1 ± 1.8
Maximum rate of rise (V/s)	8.6 ± 1.1	4.0 ± 0.7
	5.6 ± 0.6 *	3.6 ± 0.6 *
Peak potential (mV)	1.9 ± 1.3	11.0 ± 1.2
	−5.1 ± 2.4 *	8.3 ± 2.4
Duration at 50% repolarization (ms)	28.7 ± 1.3	89.3 ± 3.5
	32.2 ± 1.3 *	88.8 ± 4.2

Upper and lower values in each row indicate the parameters obtained before and after (1 min) the application of low Na^+^ solution. Values indicate the mean ± S.E.M. from 6 preparations. Asterisks indicate significant difference from corresponding values before the application of agent as evaluated by the paired *t*-test (*p* < 0.05).

**Table 4:** Action potential parameters before and after application of niflumic acid.

	Niflumic Acid	
	Mouse	Guinea Pig
Firing rate (bpm)	444.5 ± 18.1	236.6 ± 14.1
	418.4 ± 21.0 *	205.0 ± 12.5 *
Cycle length (ms)	136.1 ± 5.5	258.4 ± 15.9
	145.3 ± 7.6 *	298.0 ± 17.7 *
Maximum diastolic potential (mV)	−61.0 ± 1.1	−62.7 ± 3.1
	−60.6 ± 1.8	−61.8 ± 2.8
Slope of pacemaker depolarization (mV/s)	221.5 ± 40.9	152.2 ± 20.2
	173.8 ± 35.7 *	104.8 ± 12.4 *
Threshold potential (mV)	−50.5 ± 1.7	−48.8 ± 3.2
	−51.7 ± 2.5	−49.0 ± 3.5
Maximum rate of rise (V/s)	14.5 ± 6.5	8.4 ± 2.1
	14.7 ± 6.8	6.9 ± 1.3
Peak potential (mV)	5.8 ± 0.8	19.4 ± 4.0
	5.2 ± 1.6	17.8 ± 3.4
Duration at 50% repolarization (ms)	31.3 ± 1.2	78.5 ± 2.6
	32.1 ± 1.0	74.3 ± 3.2 *

Upper and lower values in each row indicate the parameters obtained before and after (10 min) the application of niflumic acid. Values indicate the mean ± S.E.M. from 6 preparations. Asterisks indicate significant difference from corresponding values before the application of agent as evaluated by the paired *t*-test (*p* < 0.05).

## Data Availability

Not applicable.
